# Supplementary material for: Antitumor Activities of a Humanized Cancer-Specific Anti-HER2 Monoclonal Antibody, humH2Mab-250 in Human Breast Cancer Xenografts
Source: Int J Mol Sci. 2025 Jan 26;26(3):1079. doi: 10.3390/ijms26031079 (PMC11817376; doi:10.3390/ijms26031079)
Supplement: Supplementary file 1 [file ijms-26-01079-s001.zip › ijms-3425665-supplementary.pdf]

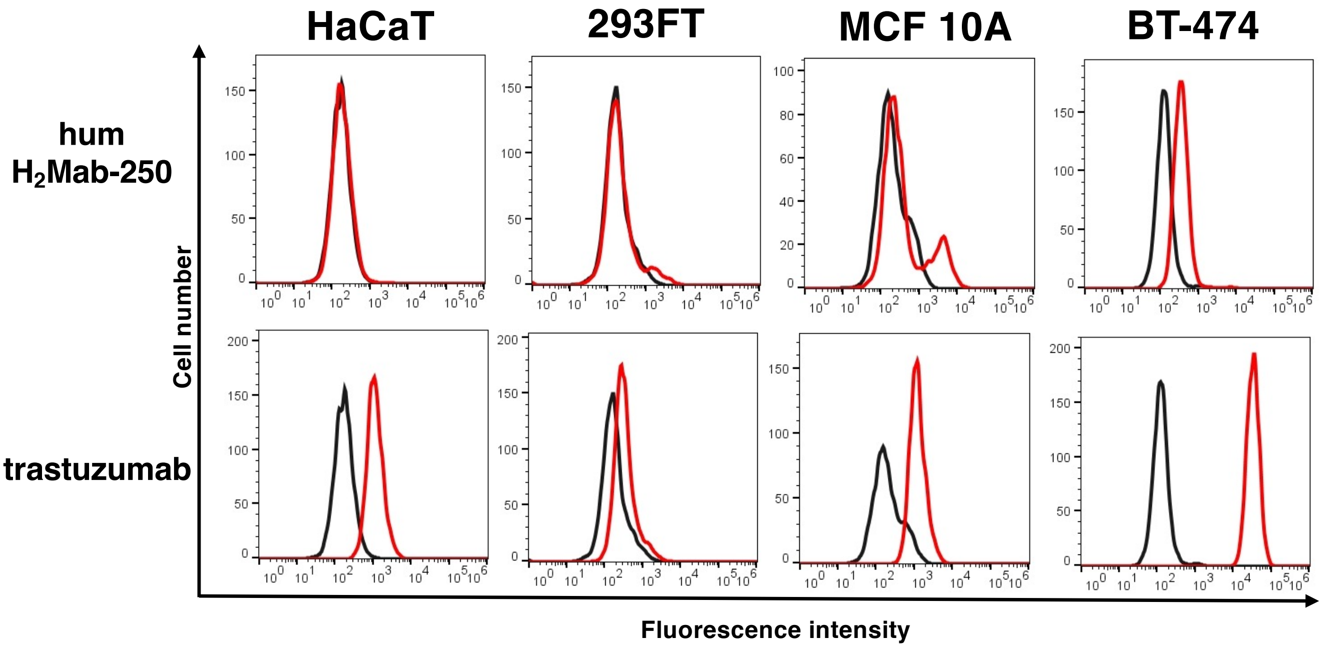

Supplementary Figure S1. Flow cytometry using humH_2Mab-250 and trastuzumab against HaCaT, 293FT, MCF 10A, and BT-474. Cells were treated with buffer control (black) or 10 μg/mL of humH_2Mab-250 or trastuzumab (red). The cells were further treated with FITC-conjugated anti-human IgG. Fluorescence data were analyzed using the SA3800 Cell Analyzer.

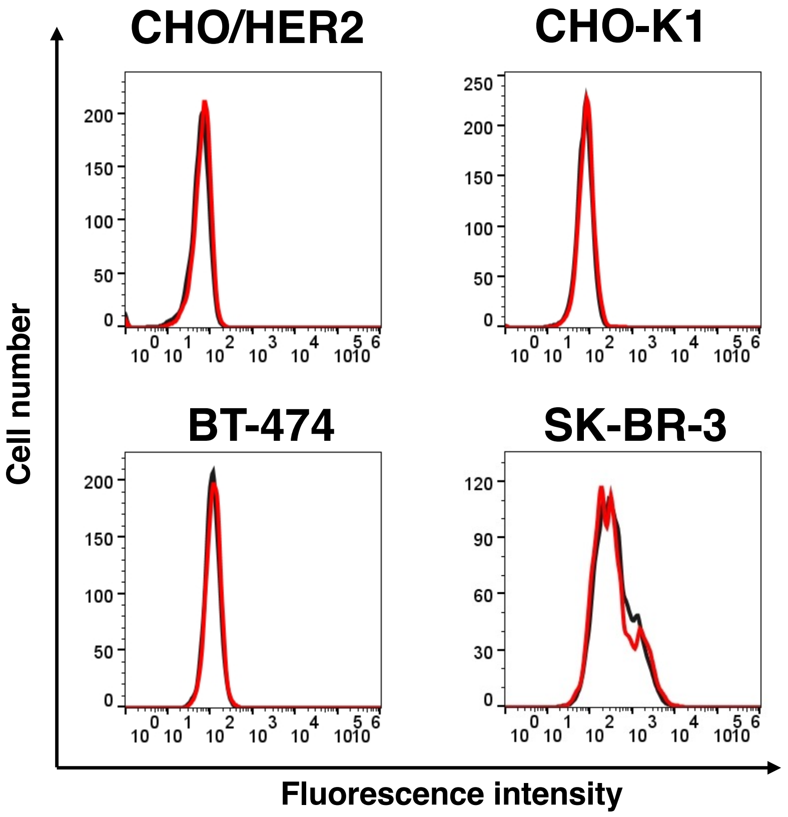

Supplementary Figure S2. Flow cytometry using humCvMab-62 against CHO/HER2, CHO-K1, BT-474, and SK-BR-3. Cells were treated with buffer control (black) or 10 μg/mL of humCvMab-62 (red). The cells were further treated with FITC-conjugated anti-human IgG. Fluorescence data were analyzed using the SA3800 Cell Analyzer.

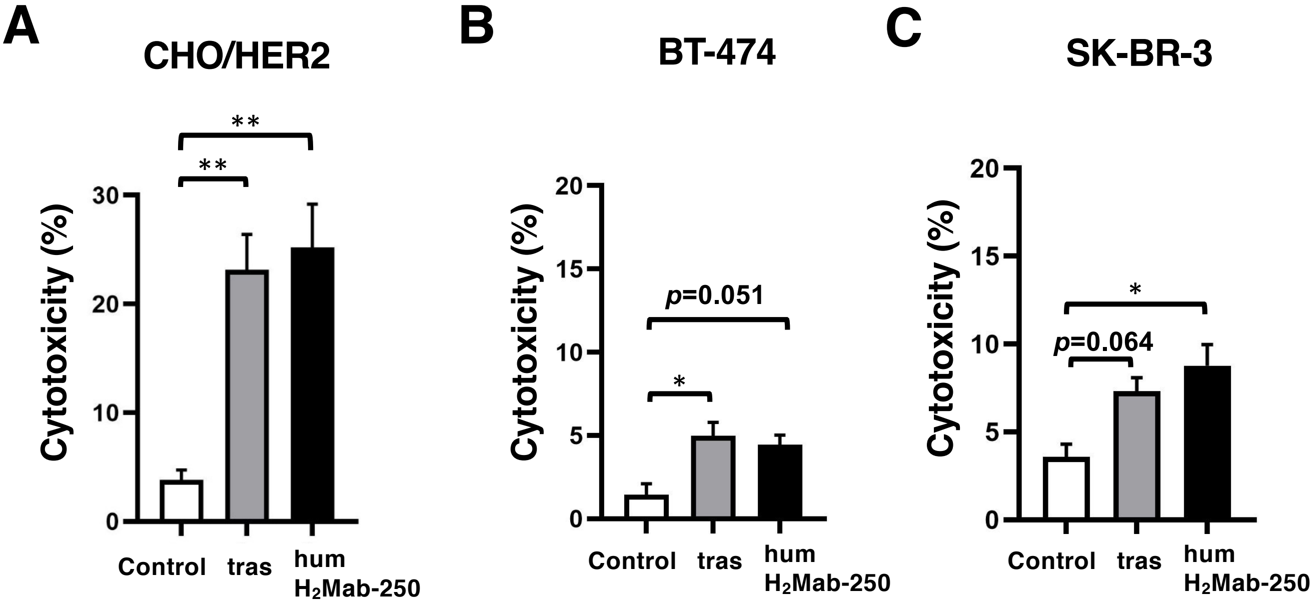

Supplementary Figure S3. The ADCC is mediated by humH_2Mab-250 and trastuzumab in the presence of human NK cells. Calcein-labeled CHO/HER2 (**A**), BT-474 (**B**), and SK-BR-3 (**C**) were treated with trastuzumab (tras), humH_2Mab-250 or control human IgG_1 in the presence of human NK cells. The cytotoxicity was determined by the release of calcein into the medium. Values are shown as the mean ± SEM. Asterisks indicate statistical significance (** $p < 0.01$, * $p < 0.05$; one-way ANOVA Tukey's multiple comparisons test).

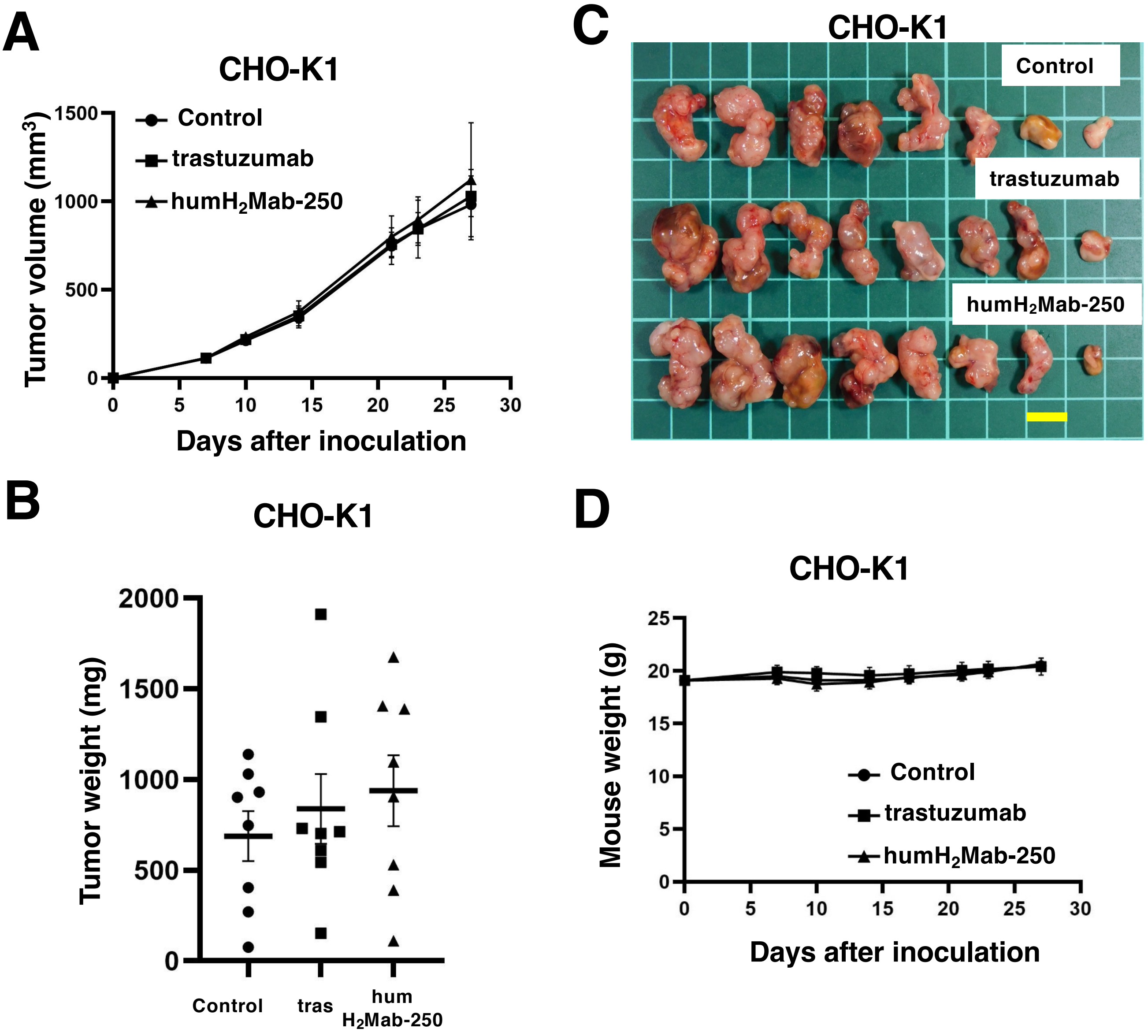

Supplementary Figure S4. No antitumor activity of humH$_2$Mab-250 against CHO-K1 xenografts. (A) CHO-K1 cells were subcutaneously injected into BALB/c nude mice (day 0). On day 7, 100 µg of humH$_2$Mab-250, trastuzumab, or control human IgG$_1$ was administered. Additional antibodies were administered on days 14 and 21. The tumor volume was measured at indicated days. (B) The xenograft tumor weight was measured on day 27. Values are shown as the mean ± SEM. There was no significant difference in Two-way ANOVA Tukey's multiple comparisons test. (C) The appearance of CHO-K1 xenograft tumors (scale bar, 1 cm). (D) Body weight of CHO-K1 xenograft-bearing mice treated with humH$_2$Mab-250, trastuzumab, or control human IgG$_1$.

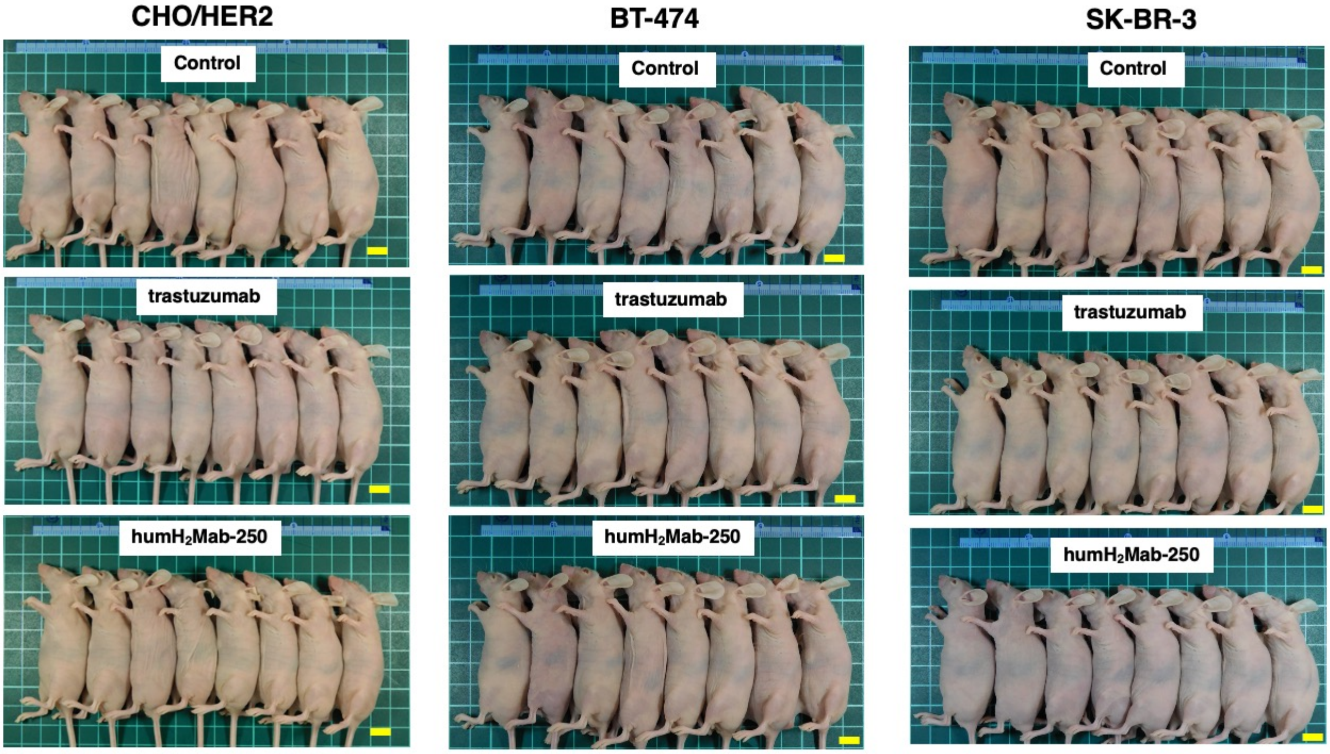

Supplementary Figure S5. Body appearance in CHO/HER2, BT-474, and SK-BR-3 xenografts-implanted mice treated with indicated mAbs. Scale bar, 1 cm.
